# FASN promotes the stemness of cancer stem cells and protects colorectal cancer cells from ferroptosis by inhibiting the activation of SREBP2

**DOI:** 10.3389/fimmu.2025.1611375

**Published:** 2025-08-18

**Authors:** Ming Wang, Fulin Ge, Cheng Wu, Binbin Su, Xiaoyu Dong, Shiping Xu, Hui Shi

**Affiliations:** Department of Gastroenterology, The Second Medical Center and National Clinical Research Center for Geriatric Diseases, Chinese Peoples Liberation Army (PLA) General Hospital, Beijing, China

**Keywords:** colorectal cancer, ferroptosis, FASN, lipid metabolism, cancer stem cells

## Abstract

**Introduction:**

Fatty acid synthase (FASN) is a key regulator of lipid metabolism, but its role in colorectal cancer (CRC) stemness and ferroptosis remains unclear.

**Methods:**

FASN expression in CRC was analyzed using TCGA data and validated in CRC cell lines (CACO-2, HCT116, SW480) and normal HIEC-6 cells via qRT-PCR and Western blot. HCT116 cells (highest FASN expression) were used for experiments. FASN silencing (shRNA) effects on CSCs were assessed via 3D spheroid formation and CD133+CD44+ flow cytometry. In vivo tumor growth was tested in BALB/c nude mice. Mechanistic assays included cholesterol detection, SREBP2 Western blot, fatostatin rescue experiments, ferroptosis markers (ferrous ions, ROS, MDA, 4-HNE, mitochondrial function), and FASN-SREBP2 co-immunoprecipitation.

**Results:**

FASN was overexpressed in CRC tissues (TCGA) and cell lines, with highest levels in HCT116. It was upregulated in 3D spheroids and CD133+CD44+ CSCs. FASN silencing reduced spheroid formation, in vivo tumor growth, and CD133+CD44+ cells. Mechanistically, FASN knockdown decreased cholesterol, activated SREBP2, and induced ferroptosis (elevated ferrous ions, ROS, lipid peroxidation, mitochondrial dysfunction); these effects were reversed by fatostatin. Co-IP confirmed FASN-SREBP2 interaction.

**Discussion:**

FASN promotes CRC progression by enhancing CSC stemness and suppressing ferroptosis through SREBP2 inhibition, highlighting its potential as a therapeutic target.

## 1 Introduction

Colorectal cancer (CRC), a neoplastic disease that originates from the epithelial cells of the mucosal lining of the colon or rectum, ranks as the third leading cause of cancer-related mortality globally, affecting over 1.85 million individuals and causing approximately 850,000 deaths annually. Recent trends indicate a notable shift towards younger populations, with rising incidence among those under 50 years (1). CRC pathogenesis involves multiple factors, including genetic predispositions (2), metabolic reprogramming (3), dysbiosis of the gut microbiota (4), alterations in tumor microenvironment (5), and cancer stem cells (CSCs) (6). As a small subpopulation with self-renewal and multipotent differentiation capacities, CSCs drive tumor initiation, progression, recurrence, and metastasis (7). Elucidating CSC-related signaling pathways and developing targeted therapies are thus critical for improving outcomes, making comprehensive investigations into CRC pathogenesis and therapeutic strategies imperative.

Ferroptosis is a regulatory form of cell death characterized by iron-dependent lethal lipid peroxides accumulation, differs morphologically, genetically, and biochemically from autophagy and necroptosis (8, 9). Cellular metabolism is central to ferroptosis: lipid peroxidation and iron buildup counteract the cysteine-GSH-GPX4 axis that suppresses this process (10, 11). During ferroptosis, polyunsaturated fatty acid oxidation in organelles combined with iron catalysis induces toxic lipid ROS accumulation (12, 13). Aberrant ferroptosis is linked to human tumors including colon cancer (14, 15). and agents like erastin reduce colon cancer cell chemoresistance and stemness via ferroptosis induction (16–18), highlighting ferroptosis induction in CSCs as a potential strategy to overcome CRC drug resistance.

Metabolic reprogramming supports both ferroptosis regulation and cancer cell survival, enabling synthesis of energy, phospholipids, and macromolecules required for growth in nutrient-deficient microenvironments (19). Lipid metabolism alterations, particularly aberrant cholesterol metabolism, promote tumorigenesis in liver cancer (20), prostate cancer (21), breast cancers (22), and CRC (23). The multienzyme protein fatty acid synthase (FASN) is a crucial modulator of lipid metabolism, particularly the production of fatty acids. In breast cancer, FASN overexpression is closely associated with tumor cell proliferation, invasion, and poor prognosis (24). High levels of FASN expression have been linked to increased fatty acid synthesis, which provides the necessary building blocks for membrane synthesis and energy storage, thereby promoting breast cancer cell growth and metastasis (25). In prostate cancer, FASN upregulation supports lipid synthesis essential for survival, with its inhibition reducing viability and inducing apoptosis (26). In terms of clinical relevance, targeting FASN has shown great potential in preclinical studies for various cancers. For instance, in breast cancer preclinical models, FASN inhibitors have been demonstrated to effectively reduce tumor growth and metastasis (27, 28). There are also ongoing clinical trials exploring the efficacy of FASN inhibitors in combination with other therapies for cancers like ovarian cancer. For CRC, despite early-stage research, FASN targeting could offer novel therapies for advanced/drug-resistant disease by disrupting lipid metabolism and inducing ferroptosis. By inhibiting FASN, we may be able to disrupt the lipid metabolism of CRC cells, induce ferroptosis, and ultimately improve patient outcomes.

The upregulation of key enzymes involved in fat production, such as FASN, ATP citrate lyase (ACLY), and several fatty acid desaturases, including stearoyl-CoA desaturase-1 (SCD1) and fatty acid desaturases 1 and 2 (FADS1 and FADS2), is thought to indicate that CSCs are highly dependent on increased fat production (29–32). Thus, by causing ferroptosis, targeting FASN appears to be a viable tactic to stop the spread of cancer. Nevertheless, the ability of controlling ferroptosis via FASN to halt the progression of CRC has not been well investigated. We specifically hypothesize that in CRC, FASN may interact with the sterol regulatory element-binding protein 2 (SREBP2) pathway. SREBP2 is a key regulator of lipid metabolism genes (33, 34). SREBP2 exists in an inactive form (f-SREBP2) bound to the endoplasmic reticulum membrane. Upon activation, it is cleaved to generate the nuclear form (n-SREBP2), which translocates to the nucleus and activates genes involved in cholesterol synthesis. We propose that FASN, under the regulation of SREBP2, promotes the synthesis of fatty acids, which in turn affects the lipid composition of cell membranes in CRC cells. This altered lipid composition may either directly or indirectly influence the sensitivity of CRC cells to ferroptosis. Additionally, FASN - SREBP2 axis may also impact the expression or activity of proteins involved in the ferroptosis regulatory network, such as GPX4, thereby modulating the overall ferroptosis process in CRC cells.

Given that lipid metabolism disruption-induced ferroptosis clearly plays a critical role in inhibiting cancer cell development, and FASN is a key molecule in lipid metabolism, the present study investigates the role and underlying mechanisms of FASN-associated lipid metabolism reprogramming in regulating ferroptosis and CSC characteristics in CRC.

## 2 Materials and methods

### 2.1 Database analysis

This retrospective study uses anonymized genomic data from colorectal cancer (CRC) patients, obtained from The Cancer Genome Atlas (TCGA) via the Genomic Data Commons Portal (https://portal.gdc.cancer.gov/). The clinical and genetic data for TCGA cohorts are registered under the dbGaP study accession number phs000178.v11.p8, with all datasets publicly accessible through the portal. The corresponding authors of this study are not involved in TCGA data sharing decisions.

We focused on two datasets: colon adenocarcinoma (COAD) and rectal adenocarcinoma (READ). The COAD dataset comprised 275 tumor samples and 349 normal tissues, while the READ dataset included 92 tumor samples and 318 normal tissues. FASN expression levels were compared between tumor and normal groups.

All analyses adhered to the Declaration of Helsinki and the International Ethical Guidelines for Biomedical Research Involving Human Subjects. Ethics oversight for TCGA is detailed at https://www.cancer.gov/about-nci/organization/ccg/research/structural-genomics/tcga/history/policies, and informed consent was obtained from all TCGA participants. We followed the Standards for Reporting Diagnostic Accuracy Studies (STARD) guidelines to ensure comprehensive and transparent reporting of methods and results.

### 2.2 Cell culture

CRC cell lines (CACO-2, HCT116, SW480) and normal human intestinal epithelial cells (HIEC-6) were purchased from Procell Life Science & Technology Co., Ltd. All cell lines were officially authenticated, with confirmed mycoplasma-free status and cell line identity (STR profiling). The cells were cultured in complete medium containing 10% FBS (Excel Bio, China), 90% high-glucose DMEM (Gibco, China) and 1% penicillin G-streptomycin double antibody (MACKLIN, China) at 37°C in a 5% CO_2_ atmosphere. When the cell density reached over 80%, the cells were digested with 0.25% trypsin (Gibco, China) for 1 min, terminated with 5 mL of complete medium, and passaged at a 1:3 ratio. For drug treatment, fatostatin (Sigma–Aldrich, USA) was added at 40 μM post-transfection, followed by 48 h co-incubated. 2 μM fer-1 (Sigma–Aldrich, USA) was added to the cells after transfection, and the mixture was coincubated for 24 h.

### 2.3 Cell transfection

HCT116 cells were seeded in 6-well plates at 1×10^6^ cells per well. A pLK0.1 vector for sh-FASN-mediated knockdown of lentivirus expression was constructed by GenePharma Co., Ltd. (GenePharma, China). A total of 125 μL of each group of plasmid vectors, including sh-NC, sh-FASN1 and sh-FASN2, was added to 125 μL of diluted Lipofectamine™ 3000 reagent (Invitrogen, USA) and incubated for 15 min to form the carrier–lipid complex, which was then transfected into the cells.

### 2.4 Animals and experimental treatment

All the animal experiments were approved by the Experimental Animal Welfare Ethics Review Committee. BALB/c nude mice (male, 5 weeks old) were purchased from Yangzhou University Center for Comparative Medicine (License No. SCXK (Su) 20220009) and housed under specific pathogen-free (SPF) conditions. A subcutaneous tumorigenic model was established by injecting 0.2 mL of HCT116 cells suspension (2×10^6^ cells, transfected with sh-FASN or sh-NC) into the flanks of nude mice. During the experiment, a Vernier caliper was used to measure the tumor size every three days at the same time, and the tumor growth curve was plotted. After 4 weeks of cell inoculation, all nude mice were euthanized, and tumors were photographed. The tumor tissue was carefully removed from the nude mouse body, and the final volume and weight of the tumors were measured. The tumor tissue was divided into two parts for preservation, one for low-temperature freezing at -80°C and the other for fixation with 4% paraformaldehyde solution for subsequent detection.

### 2.5 Real-time PCR analysis

The total RNA of cells cultured in a six-well plate was extracted via a Cell/Tissue Total RNA Isolation Kit V2 (Vazyme, China), and purified RNA was obtained after chloroform phase separation, precipitation, and washing. The RNA concentration and purity were detected via a Nano 600 (Jiapeng, China), and RNA samples with A260/280 ratios between 1.9 and 2.1 were obtained as follows. cDNA was subsequently obtained with a HiScript lll 1st Strand cDNA Synthesis Kit (Vazyme, China). Next, a real-time quantitative PCR system including Taq Pro Universal SYBR qPCR Master Mix (Vazyme, China) was prepared, and the results were detected via a CFX96 Touch 185555195 real-time fluorescence quantitative PCR instrument (Bio-Rad, USA) according to the following procedure: predenaturation at 95°C for 10 min, denaturation at 95°C for 15 s, annealing at 58°C for 30 s, extension at 72°C for 30 s, and a cycle count of 40; melting curve: 95°C for 15 s, 60°C for 60 s, and 95°C for 15 s. Relative primer (Shanghai Sangon Co., Ltd.) sequences were as follows: FASN (Forward: GTGTACGCCACCATCCTGAA; Reverse: CTGGTACAACGAGCGGATGA); β-actin (Forward: GGGACCTGACTGACTACCTC; Reverse: TCATACTCCTGCTTGCTGAT). The results were calculated via the 2^-ΔΔCt^ method. The expression of FASN was normalized to that of β-actin.

### 2.6 Western blot

The expression levels of FASN and SREBP2 were detected via western blot analysis. Briefly, total protein was extracted via RIPA lysis buffer (Beyotime, China), and a BCA kit (NCM Biotech, China) was used to determine the protein concentration. The loading volume was determined with 20 μg as the loading amount. SDS–PAGE was used to separate proteins, which were then transferred to PVDF membranes (Millipore, USA). Next, after being blocked with 5% skim milk powder (BD, USA), the membrane was incubated with FASN polyclonal antibody (Proteintech, China), SREBP2 monoclonal antibody (Proteintech, China) and β-actin antibody (Bioss, China) overnight at 4°C. The corresponding secondary antibodies, including goat anti-rabbit IgG H&L/HRP or goat anti-mouse IgG H&L/HRP (Bioss, China), were incubated at room temperature for 1 h the next day after the membranes were washed. Finally, a chemiluminescence imaging system (Tanon, China) was used for development, and the results were analyzed via ImageJ software for grayscale analysis.

### 2.7 CCK8 assay

Cell proliferation ability was detected via a CCK8 Kit (Bioss, China). Logarithmically growing cells were collected. After counting, the cell suspension concentration was adjusted, and the cells were inoculated into a 96-well plate at a density of 2×10^3^ cells/well. In accordance with the instructions, 10 μL of CCK8 solution was added, and the mixture was incubated with the cells for 2 h. Absorbance at 450 nm was determined via an RT-6000 microplate reader (Rayto, China). Cell proliferation was expressed as the cell survival rate. The calculation formula was as follows: cell survival rate (%) = (OD value (sample) - OD value (PBS))/(OD value (control group) - OD value (PBS)) × 100%.

### 2.8 Colony formation experiments

HCT116 cells whose FASN gene was successfully knocked down or treated with fatostatin were inoculated at a density of 1000 cells/well into a 6-well plate and cultured in a cell culture incubator for 14 d. Then, the cells were fixed with 4% paraformaldehyde (Beyotime, China) for 20 min and stained with 0.2% crystal violet (Sigma–Aldrich, USA) for 5 min. After washing and drying, the cells were photographed. The calculation formula was as follows: colony formation rate= number of clones/number of inoculated cells × 100%.

### 2.9 Wound healing assay

HCT116 cells were inoculated at a density of 1 × 10^6^ cells/well into a 6-well plate and incubated for 4 h. After adhering to the well, drug addition or transfection was performed. When the cells had grown to greater than 90% confluence, the tip of the pipette was used to press the line marked on the back of the plate vertically, causing scratches. The results were observed under a microscope, and the size of the cell scratch at 0 h was recorded, which was recorded again after 24 h of cultivation.

### 2.10 Transwell assay

One hundred microliters of the HCT116 cell suspension at a density of 1–5 × 10^5^ cells/mL was added to a Transwell chamber lined with matrix gel, and 600 μL of culture medium containing 10% FBS was added to the lower chamber. For the fatostatin drug treatment group, the same concentration of fatostatin was added to the upper and lower chambers and further cultivated in a culture incubator for 24 h. After being washed with PBS, 4% paraformaldehyde was added to fix the cells for 10 min. Then, the upper chamber was removed, the fixation solution in the lower chamber was discarded, and crystal violet staining solution was added. The small chamber was placed in the staining solution for 10 min. After dyeing, the upper chamber was washed with a large amount of water and dried naturally. Finally, the results were observed under a microscope, and photos were taken.

### 2.11 Detection of cell apoptosis

The apoptosis rate of HCT116 cells was detected with an Annexin V-FITC kit (Beyotime, China). After growing to cover the monolayer bottle wall, the digested cells were centrifuged and resuspended at 1000 rpm for 5 min. Then, preprepared 1 × annexin V binding solution was added to prepare a cell suspension with a final concentration of 1×10^6^ cells/mL. Then, 5 μL of Annexin V-FITC complex and 5 μL of PI solution were added to 100 μL of the cell suspension and incubated at room temperature in the dark for 15 min. Next, 400 μL of 1 × Annexin V binding solution was added. Finally, the fluorescence was detected within 1 h via Attune NxT flow cytometry (Invitrogen, USA), and the results were analyzed via Flow Jo software.

### 2.12 Sphere formation assay

Serum-free culture medium was prepared from DMEM/F12 (Gibco, China), 2% B27 (Gibco, China), epidermal growth factor (EGF; Sigma–Aldrich, USA), and fibroblast growth factor (bFGF; Sigma–Aldrich, USA). The cells were inoculated at a density of 1000 cells per well into a low-adhesion 6-well plate. After appropriate treatment was performed as needed, 3 mL of serum-free culture medium was added, and the mixture was incubated for 14 d. The results were observed and counted under a microscope.

### 2.13 Sorting of CD133+CD44+ cells and CD133-CD44- cells

**The** HCT116 cell suspension was centrifuged at 800 rpm for 5 min. After washing with 2 mL of PBS, the suspension was centrifuged again at 800 rpm for 5 min. After being resuspended in PBS, the cells were detected with a mouse anti-CD44/FITC-conjugated antibody and a rabbit anti-CD133/PE-conjugated antibody (Bioss, China) for detection via Attune NxT flow cytometry.

### 2.14 Detection steps for MDA, 4-HNE, total cholesterol and free cholesterol in cell supernatants

MDA, 4-HNE, total cholesterol and free cholesterol in cell supernatants were detected using respective kits. Briefly, logarithmic phase cell supernatants were centrifuged at 12,000×g (10 min, 4°C) for MDA/4-HNE or 10,000×g (15 min, 4°C) for cholesterol, then stored at -80°C. Standards were diluted: MDA (0-32 μmol/L with distilled water), 4-HNE (per kit instructions), cholesterol (0-3.2 mmol/L with diluent). Chromogenic reagents (TBA for MDA, 4-HNE chromogen, TC/FC reagents and enzyme working solution) were equilibrated to room temperature. MDA detection: 50 μL samples/standards/water + 150 μL TBA, boiled 30 min, ice-cooled 10 min, centrifuged (3,000×g, 10 min, 4°C), absorbance measured at 532 nm. 4-HNE detection: 96-well plate with samples/standards + chromogen, incubated at specified temperature, absorbance read at designated wavelength (e.g., 450 nm). Cholesterol-related detection: For total cholesterol detection, add 50 μL sample/standard/diluent (blank) and 150 μL chromogenic mixture (A+B) to each well, incubate at 37°C in the dark for 15 min; for free cholesterol detection, add 50 μL sample/standard/diluent and 150 μL enzyme working solution to each well, incubate at 37°C in the dark for 20 min, and measure the absorbance at 550 nm for both. Standard curves (concentration vs. absorbance) were used to calculate contents. Experiments were triplicated independently, with means used for analysis.

### 2.15 Detection of cellular Fe^2+^

The detection of the cellular Fe^2+^ content was performed following the instructions of the E-BC-K773-M ferrous ion colorimetric test kit (Elabscience, China). The OD value was measured at 593 nm via an RT-6000 microplate reader.

### 2.16 MitoSOX™ fluorescence staining

The HCT116 cells were washed with 200 μL of PBS. The MitoSOX™ Red reagent (MSR) stock solution and working solution were prepared as follows. First, the MSR reagent (Invitrogen, USA) was dissolved in 13 µL of DMSO to produce a 5 mM MSR reagent stock solution. Then, 5 µL of 5 mM MSR stock solution was added to 50 mL of PBS to prepare a 500 nM working solution. The cells were incubated and stained with 2 mL of 500 nM MSR reagent for 30 min. Finally, within 2 h of staining, an EVOS M5000 fluorescence microscope (Thermo Fisher, USA) was used to select absorption and emission wavelengths of 396 nm and 610 nm for imaging.

### 2.17 Detection of mitochondrial membrane potential levels

A JC-1 fluorescent probe (Beyotime, China) was used to detect the mitochondrial membrane potential in the cells. The JC-1 working solution was prepared according to the reagent kit method. After the cells were washed with PBS, JC-1 working solution was added to cover the cell surface for staining, and the mixture was incubated at 37°C for 15 min. The cells were examined via an EVOS M5000 fluorescence microscope after being washed with PBS once more, and the staining solution was removed. The mitochondrial membrane potential increases as the red/green fluorescence intensity ratio increases.

### 2.18 Detection of reactive oxygen species

The ROS content was detected via the use of a DCFH-DA fluorescent probe (Beyotime, China). DCFH-DA was diluted in serum-free culture medium at a ratio of 1:1000 to a final concentration of 10 µmol/L and incubated with the cells in a cell culture incubator for 20 min. The cells were washed three times with serum-free cell culture medium to fully remove the DCFH-DA that had not entered the cells. After the cells were digested and detached, an appropriate amount of culture medium was added to make a suspension of the cells, which were transferred to a centrifuge tube and then centrifuged at 1000 rpm for 5 min. Detection was performed by flow cytometry within 30 min at an excitation wavelength of 488 nm and an emission wavelength of 525 nm.

### 2.19 Statistical analysis

The experimental data are presented as the means ± SEMs, and all the data were processed and statistically analyzed via GraphPad Prism 8. ANOVA was used to compare the sample means of numerous groups, and t tests were used to compare the sample means of the two groups. A significant difference was indicated by a P value less than 0.05.

## 3 Results

### 3.1 Identification of FASN expression in CRC

**Figure 1:**
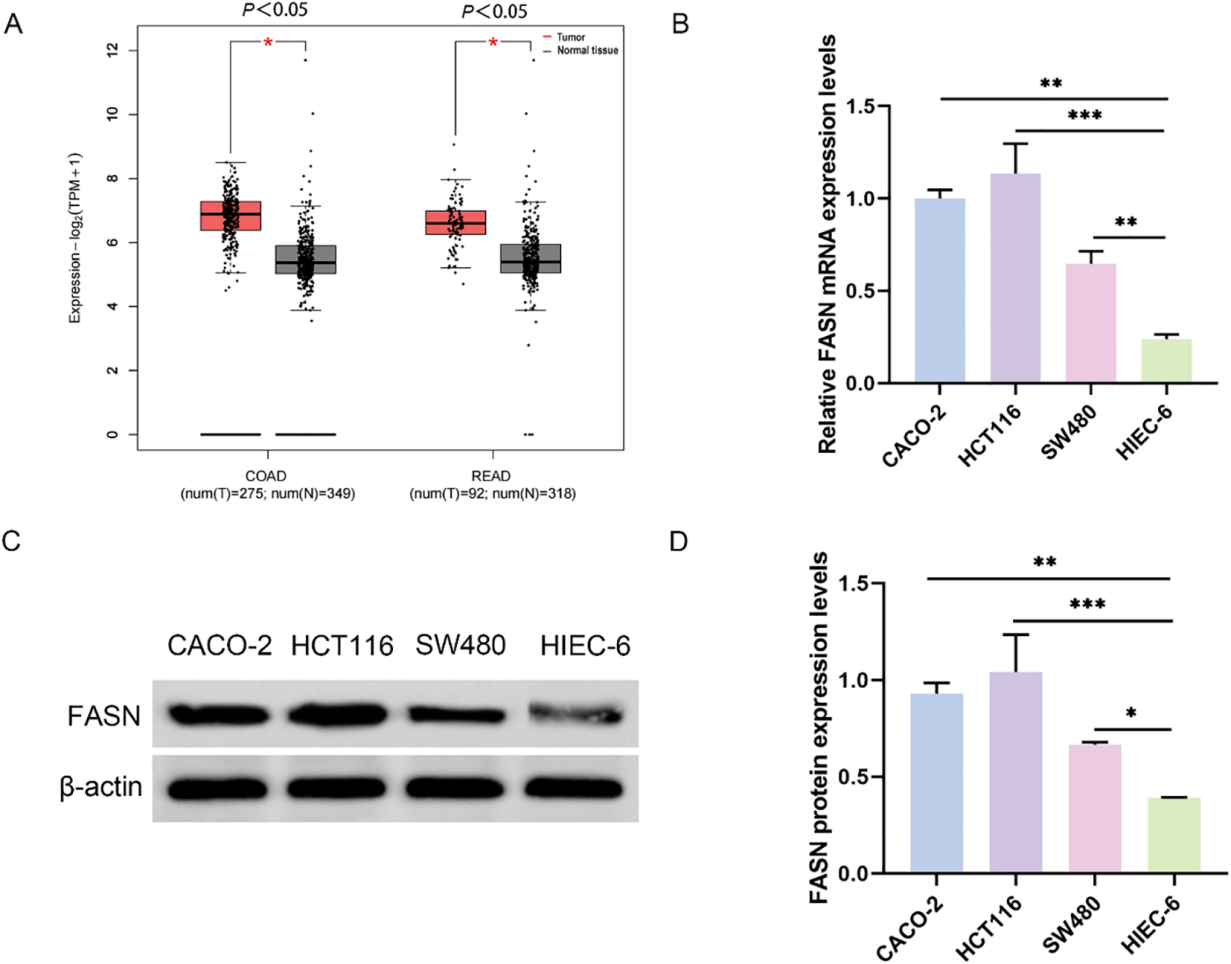
FASN expression in colorectal cancer. **(A)** Analysis of FASN expression in colorectal cancer through the TCGA database. COAD represents colon adenocarcinoma, and READ represents rectal adenocarcinoma. **(B)** Real-time PCR analysis of FASN mRNA expression levels in different colorectal cancer cell lines and normal human colorectal epithelial cells. **(C)** Western blot analysis of the protein expression levels of FASN. β-actin was used as a reference. **(D)** Quantitative analysis of the protein expression levels of FASN. ^*^ *P* < 0.05, ^**^ *P* < 0.01 and ^***^ *P* < 0.001 between the indicated groups.

Initially, we examined the expression levels of Fatty Acid Synthase (FASN) in CRC utilizing data from The Cancer Genome Atlas (TCGA) (reference 36). Our analysis revealed an upregulation of FASN expression in patients with both colon adenocarcinoma and rectal cancer, thereby providing preliminary support for our hypothesis (**Figure 1A**). To further validate these findings, we selected three CRC cell lines (CACO-2, HCT116, SW480) alongside normal human intestinal epithelial cells (HIEC-6) for additional analysis. Real-time PCR and Western blot assays demonstrated significantly elevated FASN expression in the CRC cell lines compared to HIEC-6 cells, with the highest expression observed in HCT116 cells (**Figures 1B–D**). Consequently, HCT116 cells were chosen for subsequent experiments.

### 3.2 FASN Enhances cancer stem cell characteristics and promotes tumor development in colorectal cancer

**Figure 2:**
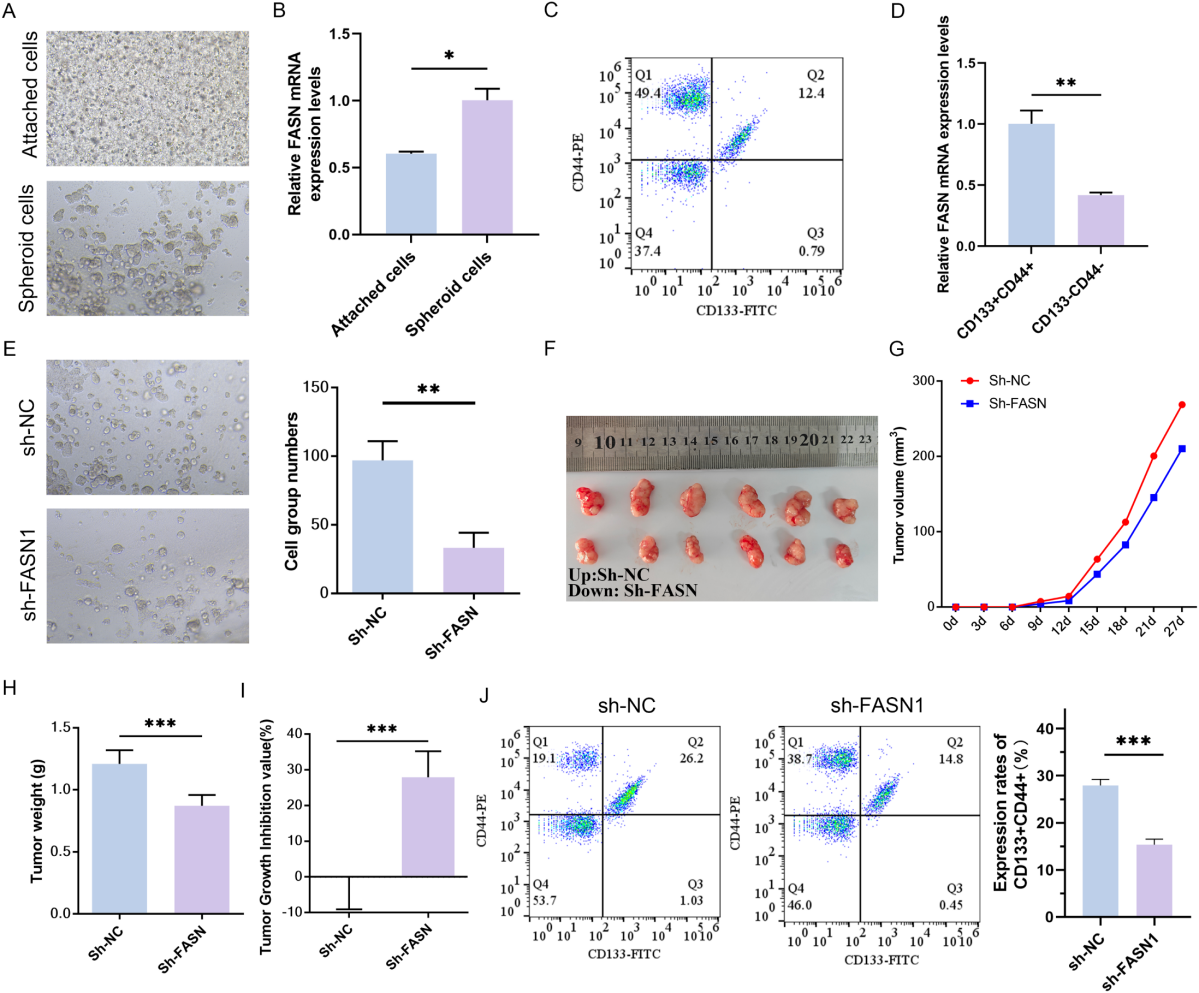
Effects of FASN knockdown on CSC characteristics in CRC cells. **(A)** Representative images of conventional culture (adherent cells) and 3D spheroidization culture (tumor spheres) of HCT116 cells. **(B)** Real-time PCR analysis of FASN mRNA expression levels in adherent cells and tumor spheres. **(C)** Flow cytometry was used to identify the CD133+CD44+ cells and CD133-CD44- cells sorted from HCT116 cells and perform quantitative analysis. **(D)** Real-time PCR analysis of FASN mRNA expression levels in different types of sorted cells. **(E)** Observation of the formation of spheroids *in vitro* after transfection with FASN-silencing plasmids and quantitative analysis. **(F)** Images of tumors from vaccinated colorectal cancer cells transfected with the sh-FASN plasmid after 4 weeks, n=6. **(G–I)** Changes in tumor volume, weight, and tumor suppression rate within 4 weeks of vaccination. **(J)** Flow cytometry was used to detect the numbers of CD133+CD44+ cells and CD133-CD44- cells and perform quantitative analysis. ^*^ *P* < 0.05, ^**^ *P* < 0.01 and ^***^ *P* < 0.001 between the indicated groups.

The development of three-dimensional (3D) tumor spheres has been employed to assess the CSC-related characteristics of solid tumors *in vitro* (37). To investigate the potential role of FASN in human colorectal CSCs, HCT116 cells were cultured using a 3D spheroidization approach (**Figure 2A**). Subsequently, real-time PCR was conducted to measure FASN expression in the tumor spheroids, revealing a significant upregulation of FASN in the spheroid cells (**Figure 2B**). Flow cytometry was then utilized to isolate CD133^+^CD44^+^ cells, indicative of CSC properties, and CD133^-^CD44^-^ cells, representative of typical cancer cell characteristics, from HCT116 cells (**Figure 2C**). Consistent with expectations, FASN expression was markedly higher in CD133^+^CD44^+^ cells (**Figure 2D**). The impact of FASN on *in vitro* spheroid formation and *in vivo* tumor transplantation models was further evaluated through FASN knockdown. Following FASN downregulation, a significant reduction in the number of spherical cells was observed (**Figure 2E**). Moreover, in the sh-FASN group, the size, volume, and weight of the tumors were substantially diminished, accompanied by an increased tumor inhibition rate (**Figures 2F–I**). Additionally, the quantity of CD44^+^CD133^+^ cells was significantly reduced as a consequence of FASN silencing (**Figure 2J**). Collectively, these results indicate that FASN is highly expressed in colorectal CSCs and is crucial for maintaining CSC characteristics, promoting spheroid formation, *in vivo* tumor growth, and preserving the CD133^+^CD44^+^ CSC population, thus contributing to CRC progression.

### 3.3 Inhibition of FASN impairs colorectal cancer progression by promoting SREBP2-mediated cholesterol metabolism

To determine the biological role of FASN in CRC cells, a sh-FASN plasmid was transfected into HCT116 cells to stably knock down FASN, and Western blot was used to verify the transfection (**Supplementary Figure S1A**). To further confirm the effect of FASN on cholesterol metabolism, we then examined the expression of sterol-regulatory element binding protein 2 (SREBP2), a key transcription factor that affects cholesterol metabolism by regulating the expression of key enzymes involved in cholesterol synthesis. In comparison with sh-NC group, the expression levels of total cholesterol and free cholesterol in the sh-FASN1 group were significantly decreased (**Supplementary Figure S1B**). Western blot assays and quantitative analysis (**Supplementary Figures S1C**) revealed that sh-FASN increased the protein expression of f-SREBP2 and n-SREBP2, while decreasing the expression of FASN. These findings indicate that FASN is associated with abnormal cholesterol accumulation in CRC.

**Figure 3:**
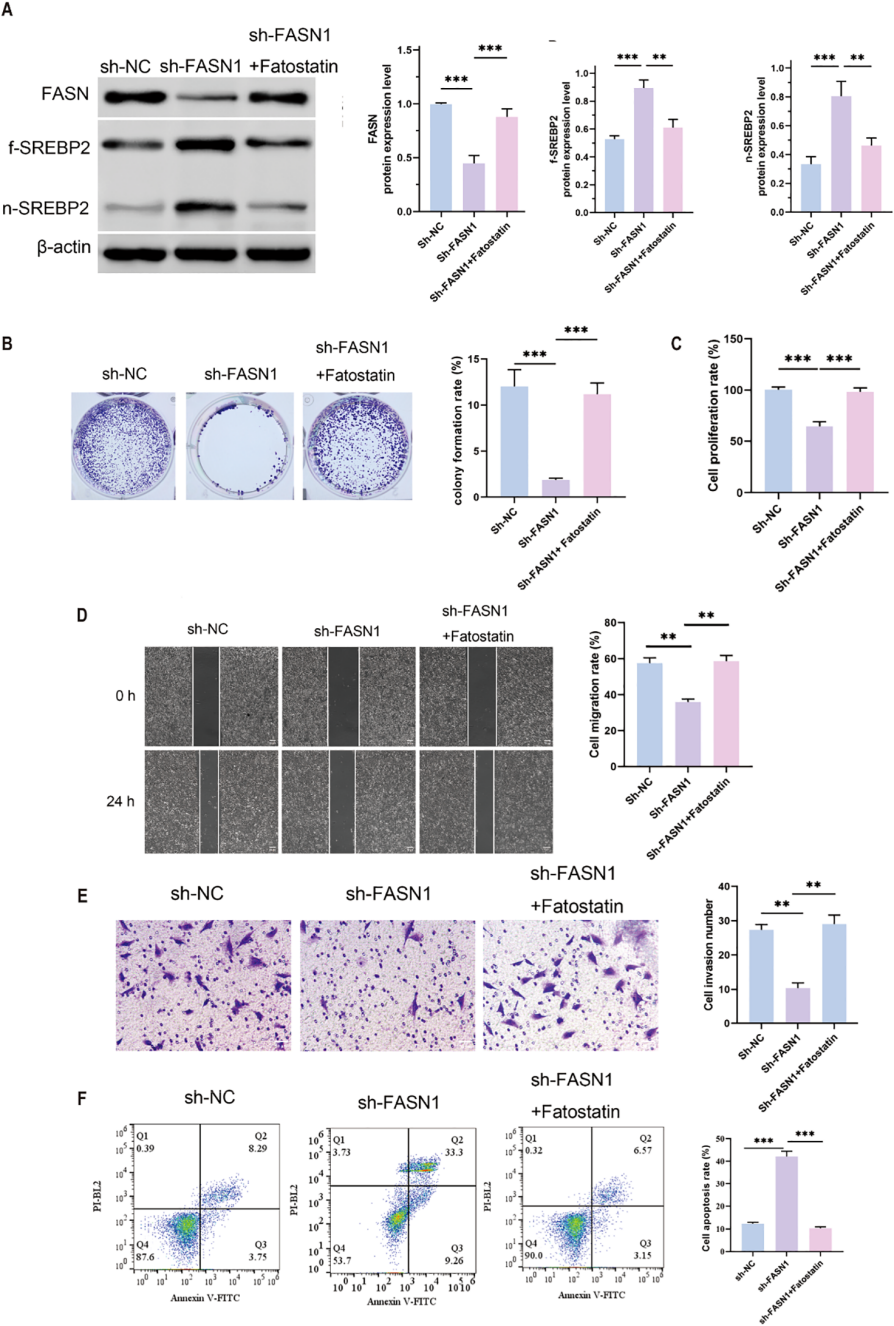
SREBP inhibition of FASN induced the malignant development of CRC cells. **(A)** Western blot analysis of the protein expression levels of FASN, f-SREBP2 and n-SREBP2. β-actin was used as a reference. **(B)** Colony formation experiments were performed to detect changes in cell growth and perform quantitative analysis. **(C)** CCK8 assay for changes in cell proliferation ability. **(D)** Wound healing assay for determining the migration ability of cells and quantitative analysis of the results. Scale bar, 50 μm. **(E)** Transwell assay for changes in cell invasion ability after FASN knockdown and quantitative analysis of the results. **(F)** Flow cytometry was used to detect the percentage of apoptotic cells, and quantitative analysis was performed. ^**^ *P* < 0.01 and ^***^ *P* < 0.001 between the indicated groups.

A series of rescue experiments utilizing fatostatin, a specific inhibitor of SREBP activation was conducted to investigate the role of FASN in CRC progression through its regulation of cholesterol metabolism. As anticipated, treatment with fatostatin resulted in a significant reduction in the protein expression levels of f-SREBP2 and n-SREBP2 forms of SREBP2, thereby confirming its inhibitory impact on SREBP2 activation (**Figure 3A**). In colony formation assays, the knockdown of FASN (sh-FASN1) led to a substantial decrease in the colony formation rate of HCT116 cells. However, co-treatment with fatostatin effectively mitigated this reduction, thereby restoring the cells’ colony-forming capacity (**Figure 3B**). Similarly, Cell Counting Kit-8 (CCK-8) assays demonstrated that sh-FASN1 significantly inhibited the proliferation of HCT116 cells, while co-treatment with fatostatin counteracted this inhibitory effect and promoted cell proliferation (**Figure 3C**). In wound healing assays, the knockdown of FASN impaired the migratory ability of HCT116 cells, but this impairment was alleviated by fatostatin, which restored the cells’ migratory potential (**Figure 3D**). Consistently, Transwell assays revealed that sh-FASN1 inhibited the invasive capacity of HCT116 cells, and this inhibitory effect was reversed upon treatment with fatostatin (**Figure 3E**). Furthermore, flow cytometry analysis revealed that FASN knockdown increased the apoptosis rate of HCT116 cells (**Figure 3F**). Taken together, these results indicate that FASN is involved in the malignant progression of CRC cells. More importantly, the ability of fatostatin to reverse the phenotypic changes induced by FASN knockdown confirms that FASN promotes CRC development by inhibiting the activation of the cholesterol metabolism regulator SREBP2, and thus inhibition of FASN impairs CRC progression by releasing this inhibitory effect on SREBP2.

### 3.4 FASN silencing induces ferroptosis via lipid peroxidation in CRC cells, counteracted by SREBP inhibition

**Figure 4:**
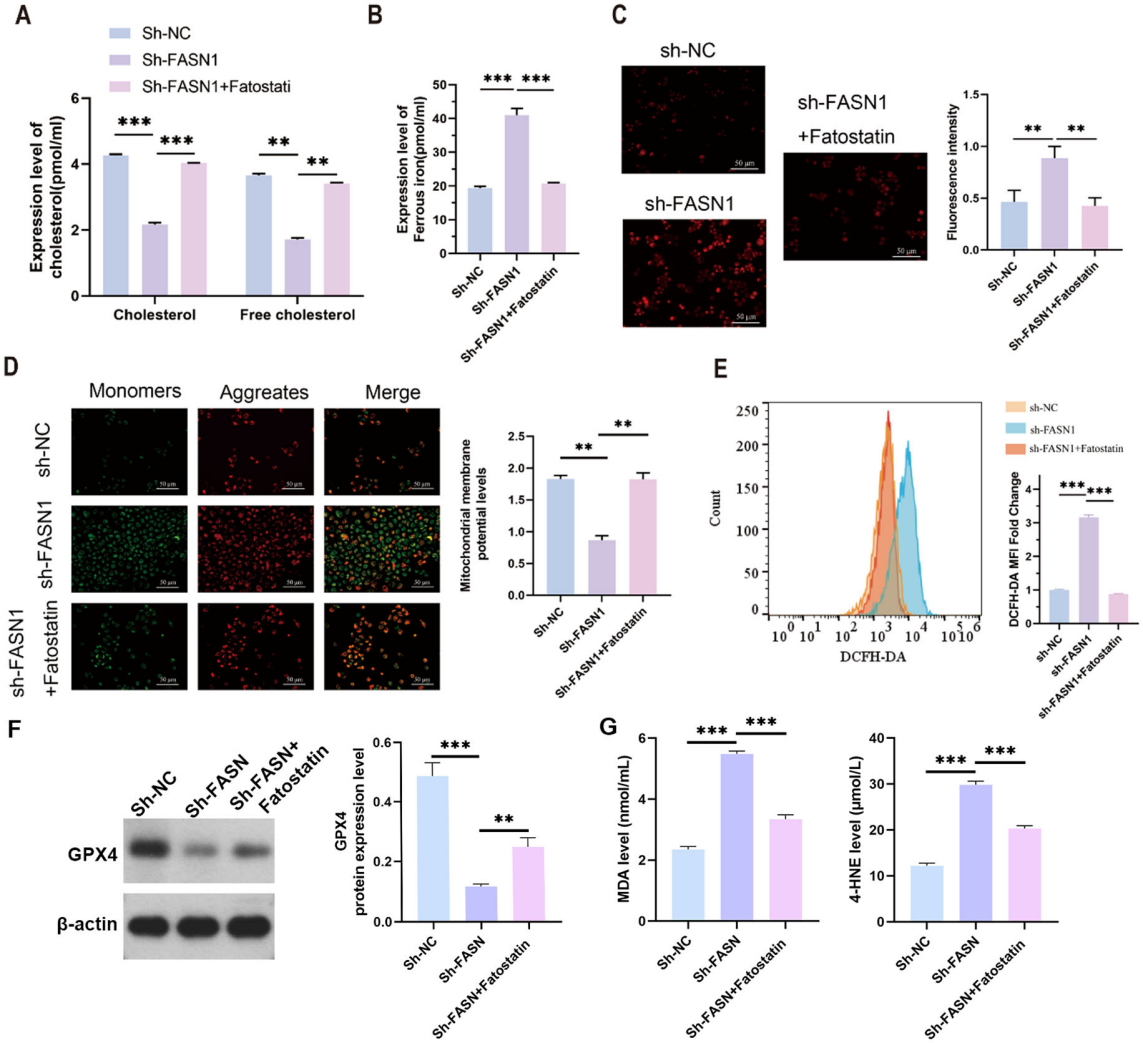
SREBP inhibition protects against ferroptosis in CRC cells. **(A)** Contents of cholesterol and free cholesterol in the colorectal cancer cells of each group. **(B)** ELISA analysis of the content of intracellular ferrous ions in each group of cells. **(C)** MitoSOX™ fluorescence staining of the intracellular mitochondrial superoxide content and its quantitative analysis. Scale bar, 50 mm. **(D)** JC-1 fluorescent probe for determination of the mitochondrial membrane potential and its quantitative analysis. Scale bar, 50 mm. **(E)** Flow cytometry analysis of the intracellular ROS content. **(F)** Western blot analysis of the protein expression levels of FASN, f-SREBP2 and n-SREBP2. b-actin was used as a reference. **(G)** Cell supernatant MDA and 4-HNE levels were measured using lipid peroxidation MDA detection kit and 4-HNE detection kit.

Both total cholesterol and free cholesterol were decreased by FASN knockdown, which were increased by fatostatin (**Figure 4A**). We then determined the effect of FASN on ferroptosis in CRC. Colorimetric assay with biochemical kits revealed that FASN knockdown elevated the level of intracellular ferrous ions, but fatostatin reduced the content of ferrous ions which was up-regulated by sh-FASN transfection (**Figure 4B**). Ferroptosis mainly occurs in mitochondria and is characterized by mitochondrial destruction. Thus, we next evaluated the influence of sh-FASN and fatostatin on mitochondrial function and oxidative stress. Sh-FASN significantly increased the content of mitochondrial superoxide in cells (**Figure 4C**). Moreover, JC-1 fluorescence probe staining revealed that sh-FASN enhanced green fluorescence and weakened red fluorescence, indicating a decrease in the mitochondrial membrane potential (MMP) (**Figure 4D**). As illustrated by flow cytometry, sh-FASN also notably increased the content of intracellular ROS (**Figure 4E**). However, fatostatin reduced the intracellular superoxide and ROS, and increased the MMP. Consistent with the ferroptosis phenotype, Western blot analysis revealed a significant reduction in GPX4 protein expression in sh-FASN compared to sh-NC. Notably, Fatostatin exhibited a partial rescue of GPX4 expression (**Figure 4F**). The malondialdehyde (MDA) content, a marker of lipid peroxidation, was significantly elevated in sh-FASN group compared to sh-NC. Conversely, sh-FASN + Fatostatin showed a marked reduction in MDA levels. The content of 4-HNE, another lipid peroxidation product, showed the same trend (**Figure 4G**). These results indicate that FASN knockdown can promote ferroptosis by enhancing lipid peroxidation, while SREBP inhibition can partially reverse this effect.

### 3.5 FASN regulates cholesterol metabolism and ferroptosis in CRC cells and directly interacts with SREBP2

**Figure 5:**
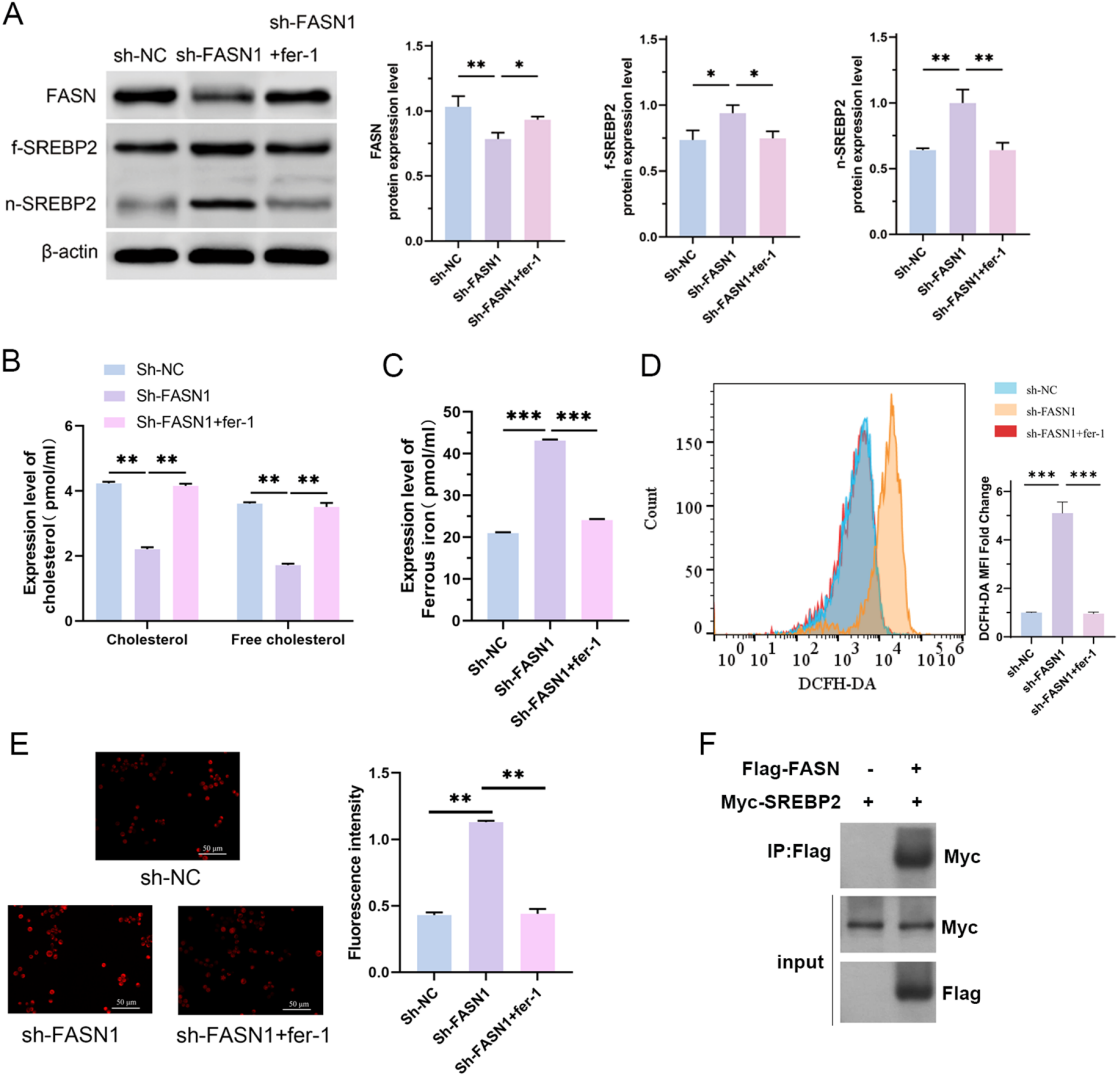
FASN regulates cholesterol metabolism and ferroptosis in CRC cells and directly interacts with SREBP2. **(A)** Western blot analysis of the protein expression levels of FASN, f-SREBP2 and n-SREBP2. β-actin was used as a reference. **(B)** Contents of cholesterol and free cholesterol in the colorectal cancer cells of each group. **(C)** Iron assay kit was used to detected the content of intracellular Fe2^+^ in each group of cells. **(D)** Flow cytometry analysis of the intracellular ROS content. **(E)** MitoSOX™ fluorescence staining of the intracellular mitochondrial superoxide content and its quantitative analysis. Scale bar, 50 μm. **(F)** Co-immunoprecipitation assays to detect the interaction between FASN and SREBP2. ^*^ *P* < 0.05, ^**^ *P* < 0.01 and ^***^ *P* < 0.001 between the indicated groups.

We conducted rescue experiments using the ferroptosis inhibitor fer-1 to further explore the relationship between FASN regulation of cholesterol metabolism and ferroptosis. As shown in **Figure 5A**, compared with the sh-FASN1 group, fer-1 treatment abrogated FASN silencing-induced changes in the expression levels of f-SREBP2, and n-SREBP2. Furthermore, fer-1 significantly attenuated FASN silencing-mediated decrease in cholesterol content in HCT116 cells (**Figure 5B**), reduction in the content of ferrous ions (**Figure 5C**), and increase in the content of intracellular ROS (**Figure 5D**) and the mitochondrial membrane potential levels (**Figure 5E**). These findings collectively demonstrate that fer-1 treatment abrogates FASN silencing-mediated alterations in cholesterol metabolism and ferroptosis-related indicators in CRC cells, confirming FASN as a critical regulatory factor for cholesterol metabolism and ferroptosis in CRC cells. Co-immunoprecipitation (CO-IP) assays were performed to investigate whether FASN directly interacts with SREBP2. HCT116 cells overexpressing Flag-tagged FASN were lysed, and the cell lysates were incubated with Flag magnetic beads. Western blot analysis of the immunoprecipitated complexes showed that SREBP2 was co-precipitated with Flag-FASN (**Figure 5F**). In contrast, no SREBP2 signal was detected in the negative control group using IgG magnetic beads, confirming the specificity of the interaction. These results demonstrate that FASN directly binds to SREBP2 in HCT116 cells.

## 4 Discussion

Mounting evidence highlights ferroptosis as a highly promising avenue for CRC therapy (35). Toxic lipids and ROS build up during the ferroptosis process as a result of the interaction between lipid peroxidation and iron catalytic activity. Unlike normal cells, tumor cells exhibit a heightened dependency on *de novo* lipogenesis, often scavenging fatty acids from the extracellular microenvironment. In the present study, we demonstrate that FASN is markedly overexpressed in CRC, and this upregulation correlates with the promotion of malignant traits such as enhanced proliferation, migration, invasion, and apoptotic resistance. Moreover, FASN appears to augment the traits of CSCs and facilitate the formation of tumor spheres. Additional investigations demonstrated that FASN confers protection to CRC cells against ferroptosis by elevating cholesterol levels and inhibiting cholesterol metabolism, which in turn fosters the abnormal accumulation of lipids within CRC cells. These findings suggest that lipid-related ferroptosis abnormalities may play an important role in CRC. However, due to the complex mechanism of its occurrence and development, further exploration is needed. In the present study, for the first time, FASN, the key regulator of fatty acid synthesis, protected CRC cells from ferroptosis by inducing cholesterol reprogramming.

Lipid metabolic reprogramming is intricately linked to CRC initiation and progression. Elevated cholesterol levels are hypothesized to fuel tumor cell proliferation, invasion, and metastasis, thereby exacerbating CRC risk. Additionally, lipid metabolic perturbations can disrupt cellular signaling cascades, impairing normal cellular functions and apoptotic machinery to promote oncogenesis (36). This is attributed to the fact that lipids can promote tumor development and metastasis by providing the energy required for proliferation (37). Our data confirm robust FASN expression in CRC, including colon adenocarcinoma, rectal cancer, and CRC cell lines. FASN, ubiquitously expressed in both plant and animal cells, catalyzes the conversion of substrates such as pyruvate and acetyl-CoA into long-chain fatty acids, which are subsequently utilized for membrane synthesis, lipid storage, or energy production (38). Thus, FASN is a pivotal player in lipid metabolism-associated disorders. To dissect FASN’s functional role in CRC, we interrogated phenotypic changes in HCT116 cells following FASN knockdown. Abrogating FASN diminished invasive, migratory, and proliferative capacities while increasing apoptotic rates. Concomitantly, FASN downregulation reduced intracellular cholesterol levels and upregulated SREBP2-a transcription factor governing cholesterol synthesis and metabolism. Upon cholesterol depletion, SREBP2 is activated, translocates to the nucleus, and transactivates genes encoding cholesterol synthetic and uptake machinery, such as HMG-CoA reductase and low-density lipoprotein receptors (39). To verify the role of cholesterol synthesis and metabolism in CRC, we specifically inhibited the activation of SREBP2 by silencing FASN. Fatostatin-induced restoration of cholesterol levels reversed the inhibitory effects of FASN knockdown on HCT116 cell phenotypes, confirming FASN’s role in orchestrating lipid reprogramming in CRC.

CSCs are postulated to be the cellular origin of CRC, driving tumorigenesis, metastasis, and therapeutic resistance (40). The uncontrolled proliferation and differentiation of CSCs, similar to the characteristics of embryonic stem cells (ESCs), leads to the occurrence and heterogeneity of tumors (41). Accumulating evidence indicates that CSCs exhibit a heightened demand for lipids; lipid metabolic pathways, including fatty acid synthesis and β-oxidation, sustain CSC survival and expansion by generating bioenergetic substrates (42, 43). Our results reveal enriched FASN expression in CD133^+^CD44^+^ CSC-like populations. FASN downregulation impaired spheroid-forming capacity and reduced the frequency of CD133^+^CD44^+^ cells, while xenotransplantation of FASN-silenced HCT116 cells attenuated tumorigenesis in nude mice. These findings reinforce the tight link between FASN-mediated lipid metabolism and CRC progression.

Lipid peroxidation, encompassing polyunsaturated fatty acids, monounsaturated fatty acids, and cholesterol, is a hallmark of ferroptosis (9). Prior studies have shown that cholesterol accumulation can sustain the expression of glutathione peroxidase 4 (GPX4), a lipid peroxidation inhibitor that counteracts ferroptosis (44). In FASN-silenced HCT116 cells, we observed an increase in the intracellular levels of iron ions, superoxide, and ROS, as well as a decrease in the mitochondrial membrane potential, all of which are markers of ferroptosis. Considering that FASN regulates cholesterol reprogramming, as mentioned above, we speculated that this may be related to a decrease in cholesterol synthesis. As expected, this was confirmed by our specific inhibition of SREBP activation. Fatostatin treatment resulted in the inhibition of ferroptosis activated by FASN silencing. Collectively, these results revealed that FASN prevents ferroptosis by affecting cholesterol synthesis and metabolism.

Given these observations, we queried whether FASN promotes CRC cell survival and proliferation by dampening ferroptosis. Inducing diverse modes of cell death, including ferroptosis, has emerged as a focal point in CRC therapeutic research (45). However, the role of ferroptosis in CRC remains controversial. Ferroptosis has been proven to be associated with colorectal epithelial diseases that can lead to cancer (46). To delineate FASN’s role in CRC ferroptosis, we performed rescue experiments using ferrostatin-1 (fer-1), a specific ferroptosis inhibitor. Fer-1 treatment elevated cholesterol levels in CRC cells, reduced intracellular iron ions, ROS, and restored mitochondrial membrane potential-consistent with a pro-survival effect.

Notably, this study has several limitations. First, our investigations focused primarily on CRC cell lines and xenograft models. While these systems provide valuable mechanistic insights, they may not fully recapitulate the complexity of human CRC, including tumor microenvironment interactions and genetic heterogeneity. Future studies should validate these findings in patient-derived xenografts (PDXs) and clinical specimens to enhance translational relevance. Our work advances the field by uncovering a novel role for FASN in ferroptosis regulation, distinct from its known functions in lipid synthesis. While prior studies have implicated FASN in CRC progression, this is the first to link FASN to ferroptosis resistance through cholesterol reprogramming, offering a unique mechanistic framework.

## 5 Conclusions

In summary, we identify FASN as a novel mediator of cholesterol synthesis and metabolism that enables CRC cells to evade ferroptosis. Our research establishes a strong correlation between high FASN expression in CRC, preservation of CSC function, and tumor progression. FASN protects CRC cells from ferroptosis by reprogramming intracellular cholesterol metabolism, thereby modulating lipid accumulation and peroxidation. These findings provide a critical theoretical basis for targeted CRC diagnosis and therapy, with significant clinical potential. However, the intricacies of lipid metabolism in malignancies necessitate further investigation: FASN may drive lipid reprogramming through other lipid species, and distinct lipids may exert divergent effects on ferroptotic pathways.

## Data Availability

The datasets presented in this study can be found in online repositories. The names of the repository/repositories and accession number(s) can be found in the article/**Supplementary Material**.

## Supplementary material

Supplementary Figure 1 FASN knockdown affects cholesterol metabolism and SREBP2 activation in CRC cells. **(A)** Western blot analysis of FASN protein expression level. β-actin was used as a reference. **(B)** Contents of cholesterol and free cholesterol in the colorectal cancer cells of each group. **(C)** Western blot analysis of the protein expression levels of FASN, f-SREBP2 and n-SREBP2. β-actin was used as a reference.
