# Supplementary figures and images for: FASN promotes the stemness of cancer stem cells and protects colorectal cancer cells from ferroptosis by inhibiting the activation of SREBP2
Source: Front Immunol. 2025 Aug 18;16:1611375. doi: 10.3389/fimmu.2025.1611375 (PMC12399527; doi:10.3389/fimmu.2025.1611375)

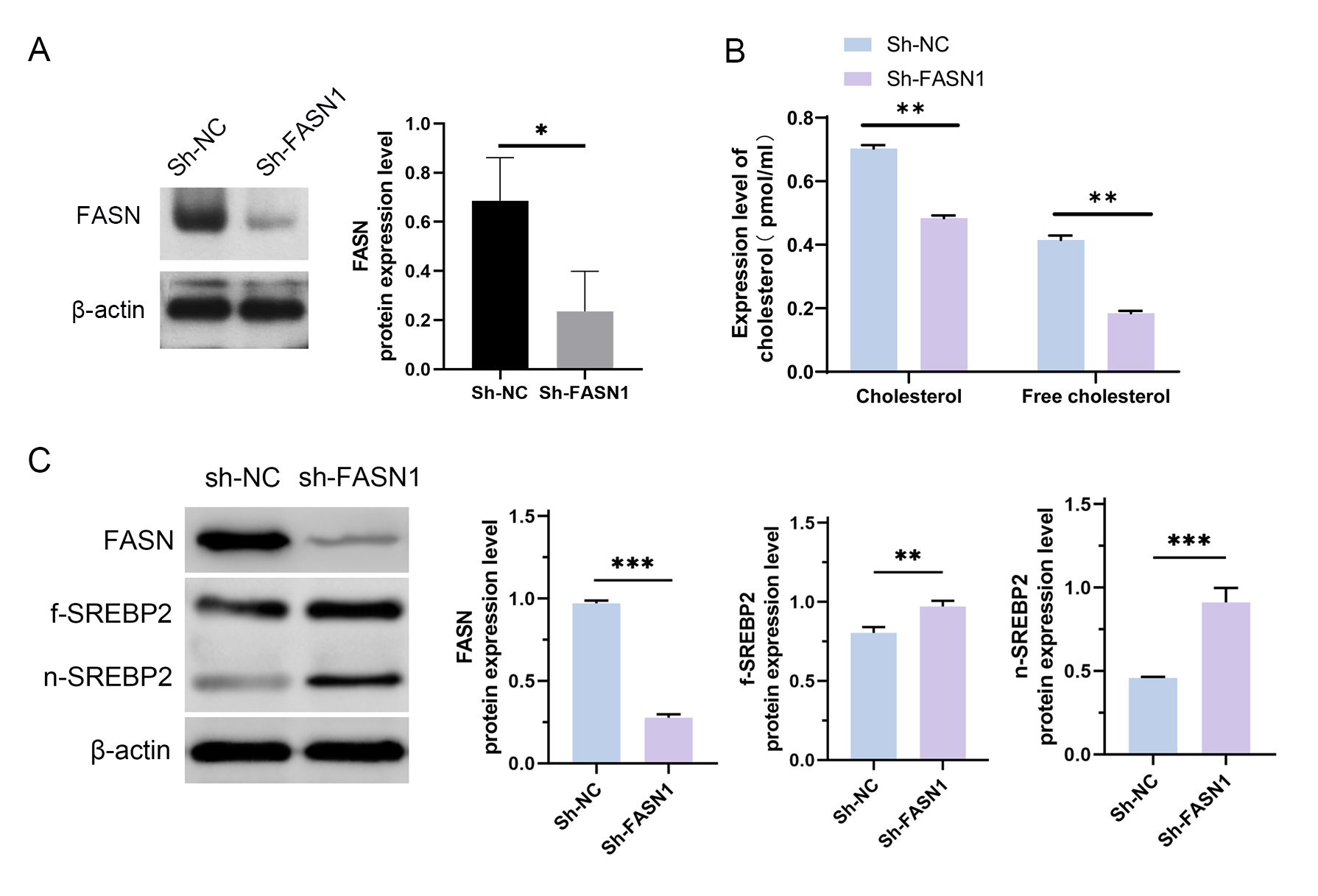

Supplement: Supplementary Figure 1 — FASN knockdown affects cholesterol metabolism and SREBP2 activation in CRC cells. (A) Western blot analysis of FASN protein expression level. β-actin was used as a reference. (B) Contents of cholesterol and free cholesterol in the colorectal cancer cells of each group. (C) Western blot analysis of the protein expression levels of FASN, f-SREBP2 and n-SREBP2. β-actin was used as a reference. [file Image1.tif]
