# Supplementary material for: FASN promotes the stemness of cancer stem cells and protects colorectal cancer cells from ferroptosis by inhibiting the activation of SREBP2
Source: Front Immunol. 2025 Aug 18;16:1611375. doi: 10.3389/fimmu.2025.1611375 (PMC12399527; doi:10.3389/fimmu.2025.1611375)
Supplement: Supplementary file 2 [file Table1.docx]

The link of the raw data: https://pan.baidu.com/s/1NKiEHh780cti9f4mKbLmzg?pwd=ieut
